# Supplementary material for: Biological Activities Underlying the Cardiovascular Benefits of Olive Oil Polyphenols: Focus on Antioxidant, Anti-Inflammatory, and Anti-Atherogenic Effects
Source: Int J Mol Sci. 2025 Nov 19;26(22):11165. doi: 10.3390/ijms262211165 (PMC12652883; doi:10.3390/ijms262211165)
Supplement: Supplementary file 1 [file ijms-26-11165-s001.zip › ijms-3918226-supplementary.pdf]

Table S1: Fatty acid composition of (EVOO+, and EVOO). Values are expressed as mean ± SEM.

		NOO		EVOO		EVOO+	
		Fatty acid (%)					
Fatty Acid Common Name	FA	Mean	esm	Mean	esm	Mean	esm
	C6:0	0	0	0	0	0	0
	C8:0	0	0	0	0	0	0
Caproic acid	C10:0	0	0	0	0	0	0
	C11:0	0	0	0	0	0	0
Lauric acid	C12:0	0	0	0	0	0	0
	C13:0	0	0	0	0	0	0
Myristic Acid	C14:0	0	0	0	0	0	0
	C14:1 t	0	0	0	0	0	0
	C14:1	0	0	0	0	0	0
	C15:0	0	0	0	0	0	0
	C15:1	0	0	0	0	0	0
**Palmitic Acid**	**C16:0**	**18,56**	**0,07**	**19,51**	**0,13**	**14,45**	**0,03**
	C16:1 t	0,16	0,00	0,15	0,00	0,09	0,00
Palmitoleic Acid	C16:1	2,08	0,00	1,90	0,01	1,23	0,00
	C17:0	0,12	0,00	0,13	0,00	0,11	0,00
	C17:1	0,23	0,00	0,24	0,00	0,17	0,00
Stearic Acid	C18:0	1,74	0,00	1,81	0,00	2,69	0,00
Elaidic Acid	C18:1 t n-9	0,00	0,00	0,00	0,00	0,00	0,00
	C18:1 t n-7	0,00	0,00	0,00	0,00	0,00	0,00
**Oleic Acid**	**C18:1 n-9**	**55,15**	**0,15**	**54,11**	**0,17**	**66,37**	**0,07**
Vaccenic Acid	C18:1 n-7	5,50	0,12	5,41	0,08	3,88	0,06
Linolelaidic Acid	C18:2 t	0,00	0,00	0,00	0,00	0,00	0,00
**Linoleic Acid**	**C18:2 n-6**	**15,29**	**0,02**	**15,55**	**0,03**	**9,76**	**0,01**
γ-Linolenic Acid (GLA)	C18:3 n-6 GLA	0,00	0,00	0,00	0,00	0,00	0,00
α-Linolenic Acid	C18:3 n-3	0,58	0,01	0,61	0,01	0,58	0,00
Arachidic acid	C20:0	0,36	0,00	0,36	0,00	0,42	0,00
	C20:1	0,24	0,01	0,23	0,00	0,26	0,00
	C21:0	0,00	0,00	0,00	0,00	0,00	0,00
	C20:2	0,00	0,00	0,00	0,00	0,00	0,00
Mead acid	C20:3 n-9	0	0	0	0	0	0
Dihomo-ɣ linolenic Acid (DGLA)	C20:3 n-6 DGLA	0	0	0	0	0	0
Arachidonic Acid	C20:4 n-6	0	0	0	0	0	0
	C20:3 n-3	0	0	0	0	0	0
Behenic acid	C22:0	0	0	0	0	0	0
	C22:1	0	0	0	0	0	0
Eicosapentaenoic Acid	C20:5 n-3 EPA	0	0	0	0	0	0
	C22:2	0	0	0	0	0	0
	C22:3	0	0	0	0	0	0
	C22:4	0	0	0	0	0	0
Lignoceric acid	C24:0	0	0	0	0	0	0
	C22:5 n-6 DPA	0	0	0	0	0	0
	C24:1	0	0	0	0	0	0
Docosapentaenoic Acid	C22:5 n-3 DPA	0	0	0	0	0	0
Docosahexaenoic Acid	C22:6 n-3 DHA	0	0	0	0	0	0
	Total	100	1,0E-14	1,0E+02	1,0E-14	1,0E+02	1,0E-14

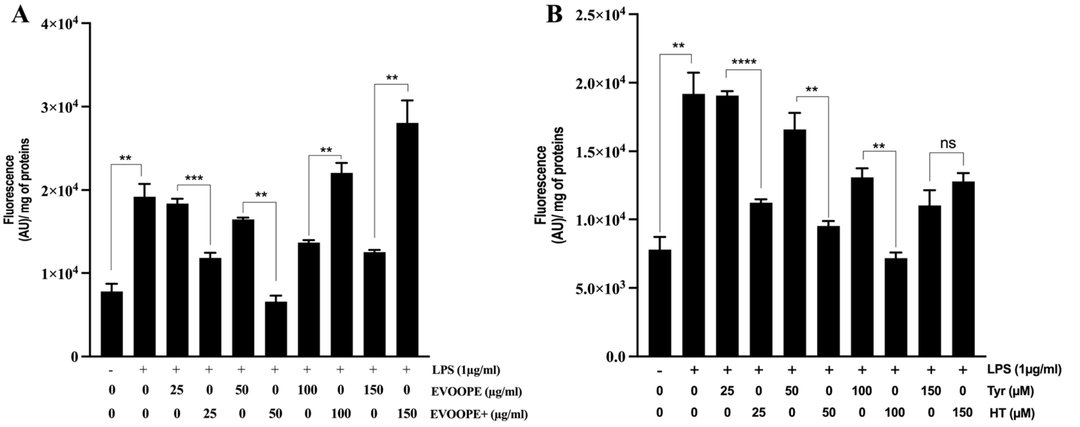

Figure S1. *(A) Comparison of the effects of EVOOPE and EVOOPE+ on ROS generation in J774 macrophages. (B) Comparison of the effects of Tyr and HT on ROS generation in J774 macrophages. Data are presented as mean ± SEM from at least three independent experiments. *p < 0.05; **p < 0.01; ***p < 0.001.*

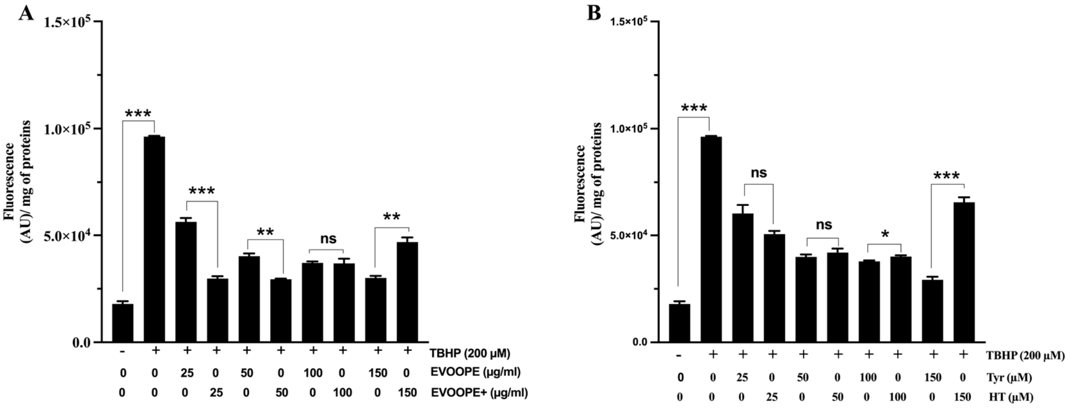

Figure S2. *(A) Comparison of the effects of EVOOPE and EVOOPE+ on Lipid peroxidation in J774 macrophages. (B) Comparison of the effects of Tyr and HT on Lipid peroxidation in J774 macrophages. Data are presented as mean ± SEM from at least three independent experiments. *p < 0.05; **p < 0.01; ***p < 0.001.*

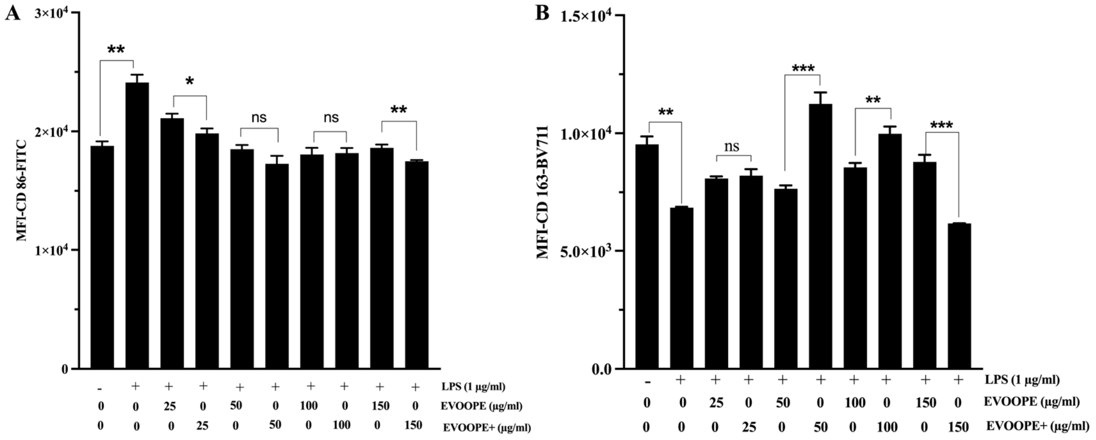

Figure S3. *(A) Comparison of the effects of EVOOPE and EVOOPE+ on CD 86 expression in THP-1-derived macrophages. (B) Comparison of the effects of EVOOPE and EVOOPE+ on CD 163 expression in THP-1 derived macrophages.. Data are presented as mean ± SEM from at least three independent experiments. *p < 0.05; **p < 0.01; ***p < 0.001.*

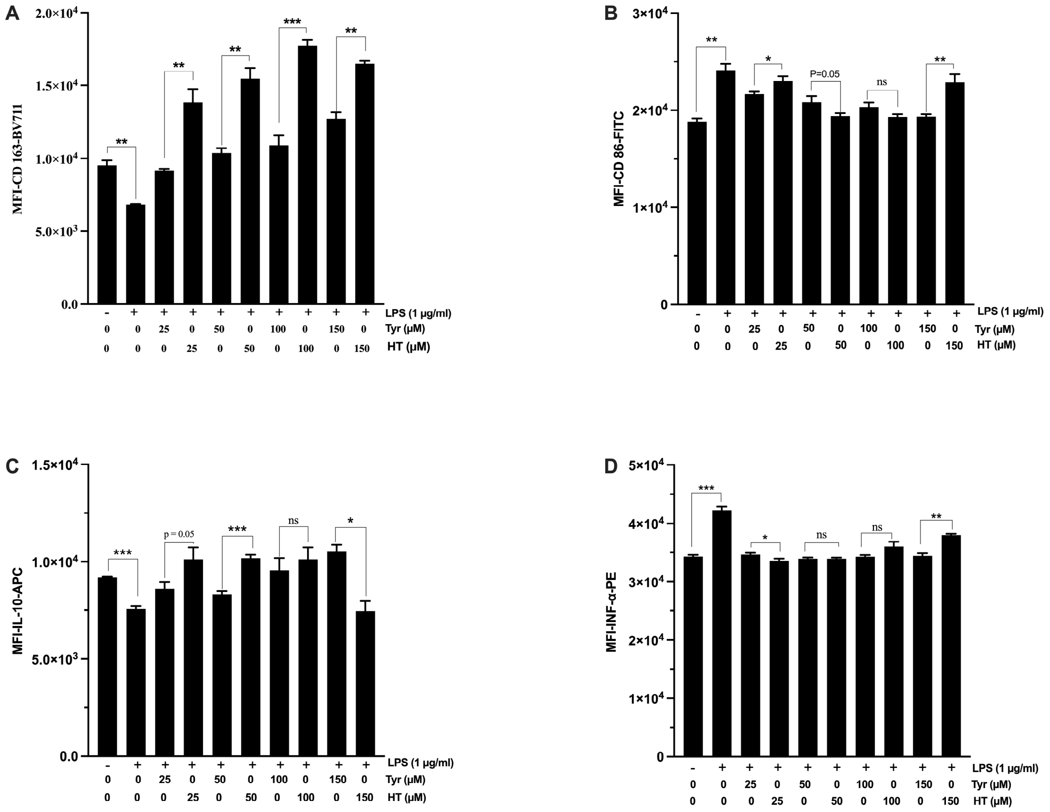

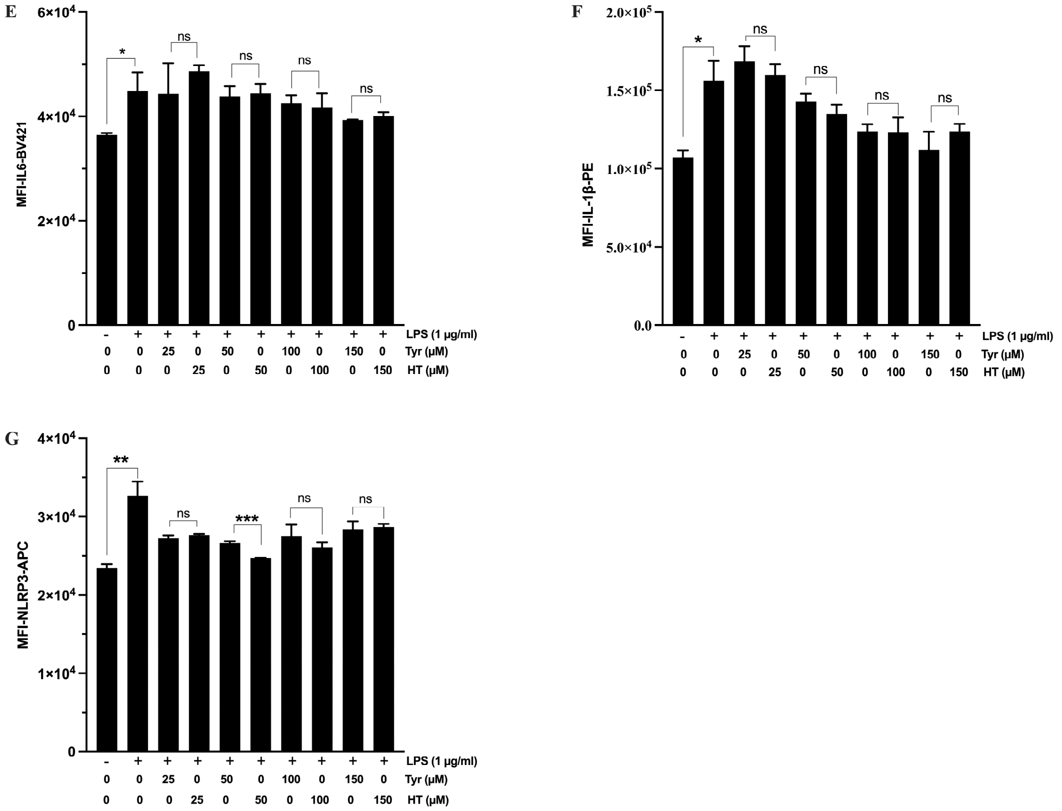

Figure S4. *Comparison of the effects of Tyr and HT on inflammatory markers in THP-1–derived macrophages: (A) CD163, (B) CD86, (C) IL-10, (D) IFN-α, (E) IL-1β, (F) IL-6, and (G) NLRP3. Data are shown as mean ± SEM from at least three independent experiments. *p < 0.05; **p < 0.01; ***p < 0.001.*

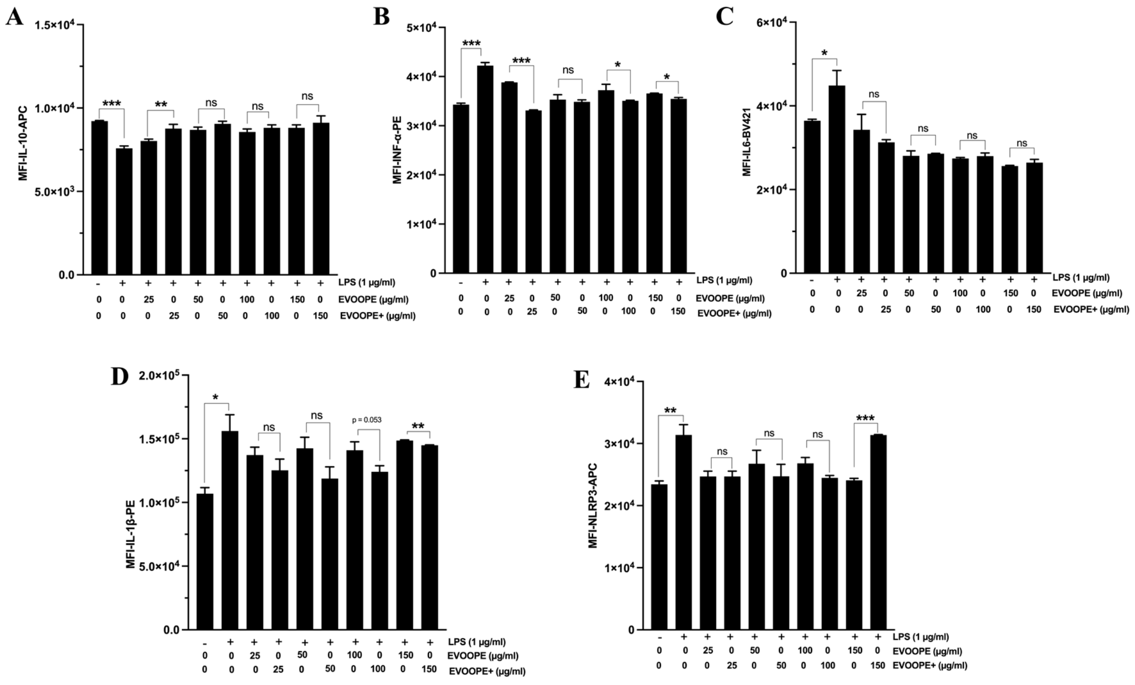

Figure S5. *Comparison of the effects of EVOOOPE and EVOOPE+ on intracellular inflammatory markers in THP-1–derived macrophages: (A) IL-10,*

*(B) IFN-α, (C) IL-6, (D) IL-1β, and (E) NLRP3. Data are shown as mean ± SEM from at least three independent experiments. *p < 0.05; **p < 0.01; ***p < 0.001.*

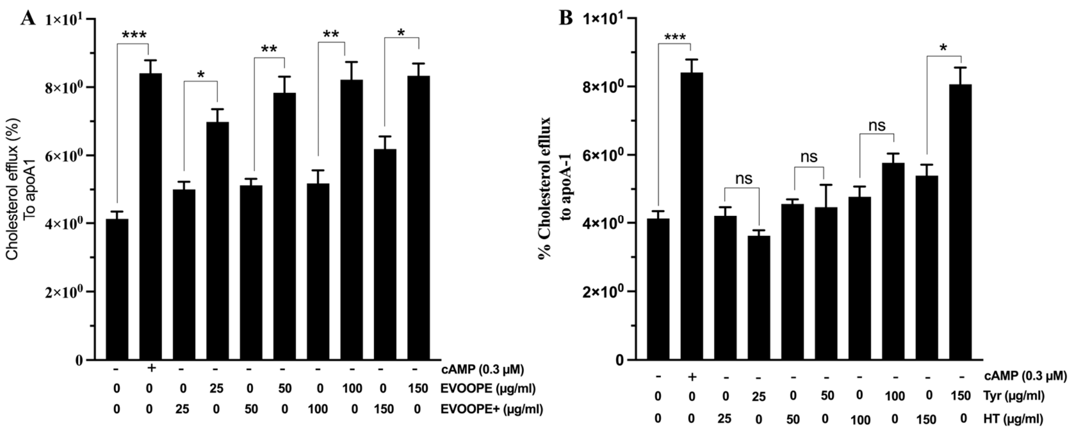

Figure S6. *(A) Comparison of the effects of EVOOPE and EVOOPE+ on Cholesterol efflux capacity in J774 macrophages. (B) Comparison of the effects of Tyr and HT on Cholesterol efflux capacity in J774 macrophages. Data are presented as mean ± SEM from at least three independent experiments. *p < 0.05; **p < 0.01; ***p < 0.001.*

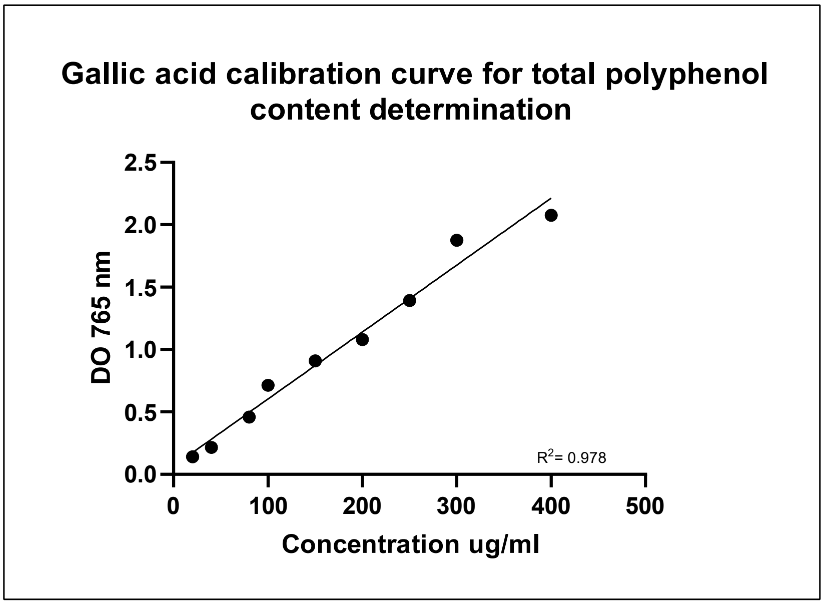

Figure S7. *Gallic acid calibration curve for total polyphenol content determination.*
